# IDOMAL: the malaria ontology revisited

**DOI:** 10.1186/2041-1480-4-16

**Published:** 2013-09-13

**Authors:** Pantelis Topalis, Elvira Mitraka, Vicky Dritsou, Emmanuel Dialynas, Christos Louis

**Affiliations:** 1 Institute of Molecular Biology and Biotechnology, Foundation for Research and Technology-Hellas, Heraklion, Crete, Greece; 2 Institute of Molecular Biology and Biotechnology, Foundation for Research and Technology-Hellas and Department of Biology, University of Crete, Heraklion, Crete, Greece; 3 Centre of Functional Genetics, Medical School, University of Perugia, Perugia, Italy

**Keywords:** Malaria, Vector borne disease, IDO, Remedies, Ontology

## Abstract

**Background:**

With about half a billion cases, of which nearly one million fatal ones, malaria constitutes one of the major infectious diseases worldwide. A recently revived effort to eliminate the disease also focuses on IT resources for its efficient control, which prominently includes the control of the mosquito vectors that transmit the Plasmodium pathogens. As part of this effort, IDOMAL has been developed and it is continually being updated.

**Findings:**

In addition to the improvement of IDOMAL’s structure and the correction of some inaccuracies, there were some major subdomain additions such as a section on natural products and remedies, and the import, from other, higher order ontologies, of several terms, which were merged with IDOMAL terms. Effort was put on rendering IDOMAL fully compatible as an extension of IDO, the Infectious Disease Ontology. The reason for the difficulties in fully reaching that target were the inherent differences between vector-borne diseases and “classical” infectious diseases, which make it necessary to specifically adjust the ontology’s architecture in order to comprise vectors and their populations.

**Conclusions:**

In addition to a higher coverage of domain-specific terms and optimizing its usage by databases and decision-support systems, the new version of IDOMAL described here allows for more cross-talk between it and other ontologies, and in particular IDO. The malaria ontology is available for downloading at the OBO Foundry (http://www.obofoundry.org/cgi-bin/detail.cgi?id=malaria_ontology) and the NCBO BioPortal (http://bioportal.bioontology.org/ontologies/1311).

## Findings

### Background

Although eradicated from most of the non-tropical regions of the world since decades, malaria is still being considered as one of the major scourges of mankind, affecting hundreds of millions of people in the tropical regions of the world [1]. Recent years have witnessed a revival of the idea of eradicating the disease, although this time the prevailing goal is that of elimination, rather than to completely expunge it [2,3]. To achieve this objective emphasis has to be put on disease control, aiming at both the disease as such (prevention, diagnosis and treatment) and, most importantly, at vector control [1]. On both fronts, different measures have to be chosen and actions such as vaccine and novel antimalarial drug development, innovative strategies for vector control and vector population monitoring, etc., have to be prominently assisted by approaches based on Information Technology (IT). It becomes clear that there is a need for new effective tools that will be able to combine different, yet related datasets covering various aspects of disease (e.g. epidemiological and entomological data, intervention efforts, etc.). These tools encompass resources such as smart databases (including decision support systems), enhanced bioinformatics software and usage of technologies such as the Internet and mobile telephony for the fast transfer of data. The latter is especially crucial, given that malaria usually strikes the world’s poorest areas, in countries in which general infrastructures are often under-developed.

It is now established that ontologies help overcome several difficulties encountered in the wide usage of IT resources by achieving enhanced interoperability. This is the reason why we decided to put emphasis on the development of ontologies that cover the domains of both vector borne diseases, including malaria, and the vectors that transmit their pathogens. We have therefore developed a series of ontologies [4] that describe the anatomy of mosquitoes and ticks, mosquito insecticide resistance, as well as malaria as the first disease in this category. It was early on decided that the latter ontology, IDOMAL [5], would be built as an extension to IDO, the Infectious Disease Ontology [6]. The first version of IDOMAL was made publicly available three years ago, at a time when IDO was still at a very early stage of development. This “wrong order” obviously led to some discrepancies between the two ontologies, which would have to be reconciled if IDOMAL is to be considered a *bona fide* extension of IDO. It is particularly important to achieve the status as early as possible, since we are in the process of developing additional ontologies in the domain of vector borne diseases such as, for example, IDODEN, a yet unpublished ontology for Dengue fever [7]. IDODEN follows the same architecture as IDOMAL, something that proved to be extremely efficient in terms of the ontology design. We therefore decided to restructure IDOMAL at this point in order to avoid major future “repair” work on several ontologies. Here, we describe both the changes introduced in IDOMAL for this purpose, as well as several other changes that have been made.

### Updating IDOMAL

All significant changes introduced to IDOMAL are indicated in Table 1. In total 222 terms were added, while another 207 changes of different nature were performed. All terms and relations that are included in the ontologies described here are in italics. The first piece of update is the conversion of IDOMAL from the OBO format to OWL. The advantages and shortcomings of the one versus the other format are not to be discussed here. Given, though, the increased spread of the OWL format among bio-ontologies we decided to proceed with the conversion, at the same time making both the OBO and the OWL formatted versions available to the community. We used the OBOtoOWL script that we previously described [8] to achieve the conversion.

**Table 1:** List of changes and corrections introduced in IDOMAL

**Update element**	**Action**	**Comments**
Non availability of IDOMAL in OWL Format	Used *OBOtoOWL* script to generate	Both versions publically available
Several terms lacked “*is_a*” paths to top	Completed “*is_a*” paths for all terms	
Multiple (11) root terms	Fully adopted BFO hierarchy to top-level	*entity* is now the single root term.
Availability of terms in other ontologies	Imported (merged) terms from other ontologies	
“Missing” terms	146 terms added	
Absence of terms relating to natural products/remedies	76 terms added (included in those above)	
Minor problems (typos, individual terms, definitions)	Edited/ completed	
Discrepancies with IDO		See Table 2 for details

The next change introduced to IDOMAL could be called technical since it concerned the editing of the ontology in order to make sure that all terms of the ontology have complete *is_a* paths to the root, which, now, in accordance to BFO (Basic Formal Ontology) [9-11] is *entity*. Therefore the term *role* which was one of many root-level terms in previous versions, now took its proper place and is to be found under *realizable entity,* which is a *specifically dependent continuant*, which is a *dependent continuant*, which is a *continuant*, which is an *entity* – the root term of BFO. Furthermore, *condition*, which was also a top-level term, is now a sibling of *role*, in accordance to the BFO 1.0 specification [9-11]. Finally, we corrected typos that were found, and added a small number of terms that had either missed our attention or their inclusion was deemed necessary given the latest developments in malaria research.

A further major addition to IDOMAL concerns the inclusion of terms from the sub-domain of natural remedies and medicinal plants. Chemotherapy of malaria has been increasingly hindered by the development of resistance of *Plasmodium* parasites against antimalarial drugs [12]. The search for novel antimalarials, therefore, has now also turned its attention to traditional remedies, in particular natural products derived from plants. It should be stressed that, currently, one such product and its derivatives (artemisinins), against which widespread resistance had not been developed, are now under such risk [13]. To cover the domain we used, mostly, a handbook [14] that fully covers the domain. We should underline the fact that we concentrated, obviously, on terms that described substances and procedures for which a certain degree of efficacy had been previously shown. Similar to what is true for “conventional” antimalarials, we do not consider the terms included as complete, and we are planning to perform more additions if necessary.

In addition to this important addition we also decided to perform a wide “exchange” of terms. The decision was taken based on the idea that in the now rapidly expanding field of bio-ontologies the same term is often defined differently, and is also linked with *is_a* relations to terms that are different. For example, querying the Bioportal [15] one finds that the term *symptom* is now described in 15 distinct ontologies. Interestingly, IDO includes *symptom* as a *quality* and has no children terms, the Ontology for General Medical Science (OGMS) [16] has *entity* as its parent and only lists one child (*pain*) while, finally, the Influenza ontology (FLU) [17] also has *entity* as the parent of symptom (also imported from OGMS) and has 8 children listed that, though, do not include *pain*. It should be noted that FLU is an extension of IDO. In IDOMAL, *symptom* (with a large number of children) is linked to *condition of the malaria host*, which is obviously a child of *condition*. We decided to replace all children of *symptom* with those listed by the Symptom Ontology (SYMP) [18], in which *symptom* is the root. By term replacing we actually mean the merging of the terms from SYMP to those in IDOMAL. Merging, instead of replacing leaves both IDs intact within IDOMAL, and therefore if somebody has already been using IDOMAL there will be no need to perform any changes in the software that uses the ontology. The choice of SYMP was made purely on the fact that alternatives such as OGMS do not list the terms that we needed. Finally, we should state at this point that IDO is using the *symptom* term imported from OGMS (see below).

### IDOMAL and IDO

As mentioned earlier, due to the timing of development of the two ontologies the published version of IDOMAL has some features that make it difficult, as such, to be called an extension of IDO in the latter’s present form. The example stated above, i.e. the term *symptom* being imported from two different ontologies, exemplifies this problem while, at the same time, it also shows that the differences are not necessarily irreconcilable: the easiest solution for this kind of discrepancy would be to simply merge the two terms. There are several more examples of how some differences may be eliminated. Table 2 lists these, showing in addition the actions taken or to be taken. For example, while *antiparasitic drug* is a role in IDOMAL, a term *antiparasitic disposition* is found in IDO, defining *antiparasitic material entity* as entity, which bears antiparasitic disposition (IDO contains dispositions such as antibacterial, antifungal, antiparasitic, antimicrobial, antiviral). We could easily reconcile the difference by accepting that *antimalarial drug* in IDOMAL is a role borne by a *material entity* which has *antiparasitic disposition* and is given to a patient to treat malaria. This would follow the example set in IDO by *antiseptic role* (definition: A role borne by a material entity in virtue of the fact that it has an antimicrobial disposition and is applied to an anatomical entity of a living organism). There are a few more cases of discrepancy between the two ontologies and for some of them we have decided to adopt the IDO point of view. For example, *endemicity* that was a *disposition* in IDOMAL will be changed to *quality of a population*, and so will be the terms *holoendemicity*, *hypoendemicity* and *mesoendemicity*, which are absent from IDO. We have also changed *resistance* from *quality* to *disposition*; although a good case was made for the fact that *resistance* is a *disposition*[19], we should nevertheless state here that resistance is in most, if not all cases a genetic phenotype. And without going into further discussions, we simply state that phenotypes are usually considered to be *quality*, possibly because of the fact that several of them are visible (*e.g.* white eyes, ectopic expression, etc.). Finally, *habitat*, a *spatial region* so far in IDOMAL, has been changed to *site* like in IDO. What also had to be changed to fit the present ontological representation is to define *breeding site* as a *role* carried by *material entity* (i.e. *site*).

**Table 2:** Differences between IDO and IDOMAL and steps (to be) taken to unify the ontologies

	**IDO-IDOMAL “comparison”**	**Action**	**Comments**
IDO:	*symptom* imported from OGMS, no children		
IDOMAL:	native term, 39 children	Imported/merged terms from Symptom Ontology	
IDO:	*endemicity* is *quality of a population*		
IDOMAL:	*endemicity* is *disposition*	Changed in IDOMAL, now as in IDO	
IDO:	*habitat* is *site* (material entity)		
IDOMAL:	*habitat* is *spatial region*	Changed in IDOMAL, now as in IDO	
IDO:	*breeding site* absent		
IDOMAL:	*Anopheles breeding site* is a *role*	Changed to Anopheles breeding site role born by site (material entity)	
IDO:	*epidemiological types* [of an infectious disease] absent		
IDOMAL:	*epidemiological types of malaria* present	Discuss with IDO developers	Needed in IDOMAL
IDO:	*antiparasitic material entity* is *material entity* bearing *antiparasitic disposition*		
IDOMAL:	*antiparasitic drug* is a *role*.	Issue solved in IDOMAL: antimalarial drug is a role born by a material entity which has antiparasitic disposition	Similar for antibiotic role/ disposition, etc.
IDO:	*resistance* is a *disposition*		
IDOMAL:	*resistance* is *quality* (of (*vector population*)	Changed in IDOMAL, now as in IDO	
IDO:	*infection* is a *material entity*		
IDOMAL:	*infection* is not present; *infectious disposition* is not present	No action needed	Terms are not needed in IDOMAL
IDO:	*vector competence* is not present		
IDOMAL:	*vector competence* is present	Discuss with IDO developers	Needed in IDOMAL
IDO:	*infectious disease control* is absent		
IDOMAL:	*malaria control* is present	Discuss with IDO developers	Needed in IDOMAL
IDO:	No terms for decision support systems, [disease] and vector control, intervention methods		
IDOMAL:	Corresponding terms present	Discuss with IDO developers	Needed in IDOMAL
IDO:	*refractoriness* is obsolete term		
IDOMAL:	*refractoriness* is present	Discuss with IDO developers	Needed in IDOMAL
IDO:	*treatment* is obsolete term in IDO		
IDOMAL:	*treatment* is present	Discuss with IDO developers	Needed in IDOMAL
IDO:	*holoendemicity* / *hypoendemicity* / *mesoendemicity* are missing		
IDOMAL:	terms are present	Discuss with IDO developers	Needed in IDOMAL
IDO:	*quality of infectious disease* is absent		
IDOMAL:	*quality of malaria* is present	Discuss with IDO developers	Needed in IDOMAL, acts as a placeholder for terms relating to malaria-specific interventions.
IDO:	*process* of infectious disease is absent		
IDOMAL:	*process of malaria* is present	Discuss with IDO developers	
IDO:	no differentiation between host/patient and pathogen		
IDOMAL:	differentiates between host/patient, vector and pathogen	Discuss with IDO developers	Needed in IDOMAL, coverage of physiological and pathophysiological processes occurring in host and/or vector and/or parasite.

In spite of the changes made, a series of issues remain that haven’t yet been resolved. Some of them, in our opinion, are relatively secondary and they could be resolved easily. For example, terms such as *treatment* and *refractoriness* which we deem to be necessary for an ontology of vector borne diseases could be carried again by IDO, from which they were obsoleted some time ago.

The remaining open issues are due to the distinctive properties of vector borne diseases. These infections are characterized by the fact that they arise through the biological interactions between three organisms (patient/host, vector and pathogen), rather than only two as is common in the vast majority of infectious diseases. Thus, an ontology such as IDOMAL has to capture all three organisms, as well as prominently include terms on the respective populations. For example, control of malaria, eventually leading, perhaps, to its elimination, is predominantly based on vector control. This involves measures aimed, for example, at reducing mosquito populations, possibly using genetic approaches [20] or, as may be the case in the future if planned strategies succeed, at replacing vector populations with others that will simply not be able to transmit the pathogen [21].

Both IDO and IDOMAL use the BFO [9-11] as an upper level ontology. IDO would then describe the infectious disease domain, while IDOMAL would ideally be placed below it. The current structure of IDO, though, does not allow for a full deployment of the malaria domain. IDOMAL has separated several classes of terms in three main “groups” namely the patient/host, the vector and the pathogen or, to be more precise, into six groups since populations are “treated” separately. The reason for this is obvious: many terms apply to both patient/host and vector (both being metazoan). Similarly, the latter separation, of course, is due to the fact that several terms are specific for populations, rather than individuals; this is especially true for vector control.

Another problem that is not yet resolved is how to list in a grouped, ontologically correct form terms such as *pathogen specific form of malaria* and *epidemiological type of malaria*. A solution could be to create sub-ontologies for each one of the different forms of malaria (i.e. for *P. falciparum*, *P. malariae*, *P. ovale* and *P. vivax*); we consider this to be impractical in several obvious aspects. For the time being we keep the problematic class *quality of malaria* and we’ll aim at finding an appropriate way to describe these features in collaboration with the IDO consortium. The class *process of malaria*, thus, groups a series of physiological and pathophysiological processes occurring in the patient/host and/or the parasite. Finally, IDOMAL has no placeholders for vector-specific processes (e.g. host seeking) or qualities (e.g. vector competence). Of course, all of these terms could be listed as direct children of *process* and *quality*, but we think that a more detailed classification would benefit the users of the ontology and, especially, would make it easier to design other ontologies for vector-borne diseases. A similar consideration is valid for *malaria prevention* and *vector control*, terms that need to be included, and are now under the “place holder” *process of malaria*. It should be noted here that recently the Vector Surveillance and Management Ontology (VSMO) was published that covers the domain of vector control [22]. This development may make it easier to find a partial solution to the last mentioned problem.

## Conclusions

It was not unexpected that IDOMAL had to undergo several updates, partial revisions and expansion during the three years after it was published, which all are summarized in this report. Not only is it legitimate to always try to obtain a better “product”, but also some of the changes are dictated by the needs of the community (e.g. remedies and natural products) or recent developments in the field. As mentioned in the beginning, improved IT tools are becoming indispensable, especially as high throughput technology develops and provides more data. In the case of malaria and other vector borne diseases, this evolution is obvious. Only about ten years after the determination of the genome sequence of *Anopheles gambiae*[23] tens of genomes of different vectors have become available [24]. Although so far genes are usually only annotated with GO terms [25], the day is not far when they, and other data in genomic/biological databases, will also be annotated with ontological terms describing these domains, such as, for example, VectorBase, the database that covers arthropod disease vectors [26]. Moreover novel IT tools such as decision support systems are already making use of ontologies [27] and, even more, tools are planned that will be able to direct information to and from ontologies and data holders [28]. In the domain of vector-borne diseases, IDOMAL and MIRO, an ontology of Mosquito Insecticide Resistance [29] that has now been fully integrated in IDOMAL, are used by newly developed Decision Support Systems for vector-borne diseases [30,31]. Furthermore, VSMO also uses a several terms that have been imported from IDOMAL [22]. It becomes clear that the availability of all the new, open bio-medical ontologies provides ways to achieve enhanced interoperability between databases and to expand the title of the original publication of the Gene Ontology [32] to “tools for the unification of bio-medical sciences”.

***N.B.*** Both IDOMAL versions are available for downloading: the OBO version is at the OBO Foundry and at the NCBO BioPortal, while the OWL version is available at: http://anobase.vectorbase.org/idomal/.

## Abbreviations

BFO: Basic formal ontology; FLU: Influenza ontology; GO: Gene ontology; IDO: Infectious disease ontology; IDODEN: Infectious disease ontology-dengue; IDOMAL: Infectious disease ontology-malaria; MIRO: Mosquito insecticide resistance ontology; NCBO: National center for biomedical ontology; OGMS: Ontology for general medical science; OBO: Open and biomedical ontologies; OWL: Web ontology language; SYMP: Symptom ontology; VMSO: Vector surveillance and management ontology.

## Competing interests

The authors declare that they have no competing interests.

## Authors’ contributions

PT was responsible for the final updating process and oversaw the regular operations; EM, VD and ED were responsible for individual parts of the update project; CL researched the domain of natural products and wrote the first draft of the paper (which all other authors helped finalize) and coordinated the study. All authors read and approved the final manuscript.
